# Exogenous strigolactones modulate antioxidant metabolism via *CsD27* to enhance drought tolerance in tea plants

**DOI:** 10.3389/fpls.2025.1601094

**Published:** 2025-06-06

**Authors:** Jingyue Shen, Manni Tong, Qingyun Yuan, Lizhi Long, Yuanzhi Shi

**Affiliations:** ^1^ Key Laboratory of Tea Biology and Resource Utilization of Tea, Tea Research Institute, Chinese Academy of Agricultural Sciences, Hangzhou, China; ^2^ Graduate School of Chinese Academy of Agriculture Science, Beijing, China

**Keywords:** strigolactones, drought stress, tea plant, CsD27, catechin

## Abstract

Drought stress adversely affects the growth, yield, and quality of tea plants (*Camellia sinensis* L.). Although strigolactones (SLs) are known to mediate drought adaptation in plants, their regulatory mechanisms in tea plants remain elusive. In this study, we demonstrated that exogenous SL application alleviated drought-induced symptoms by photosynthetic adaptation and mitigating the damage of cell membrane. Moreover, exogenous SL enhanced antioxidant response through regulating catechins metabolism in drought-sensitive cv. ‘Huangjinya’. Notably, we identified *CsD27* as a key SL-biosynthetic gene, whose expression level was negatively correlated with malondialdehyde (MDA), mechanistically linking its function in drought tolerance in tea plants. Overexpression of *CsD27* enhanced the drought tolerance of transgenic *Arabidopsis* with decreased MDA content and increased survival rate under drought stress. These findings elucidate a dual SL-mediated mechanism that simultaneously enhances stress tolerance and preserves tea quality, which provide a potential target for molecular breeding in perennial crops.

## 1 Introduction

Tea plant (*Camellia sinensis* L.), a perennial crop of significant economic importance, produces the world’s second most consumed non-alcoholic beverage. This commercially important species is extensively cultivated across more than 50 countries spanning five continents (Ahmed et al., 2019). However, due to its hygrophilous nature and the spatial heterogeneity of water resources in major tea-growing regions, tea plants face increasing exposure to drought stress. In fact, drought has emerged as one of the predominant abiotic constraints on global tea production (Lv et al., 2021; Yang et al., 2024), causing reduced shoot elongation, leaf chlorosis, and progressive wilting that ultimately lead to substantial yield reduction (Liu, 2015). Furthermore, the compounding effects of severe drought extend beyond physiological damage to fundamentally alter tea’s biochemical fingerprint, triggering metabolic reprogramming that reduces key quality-determining metabolites such as catechins, caffeine, theanine, and certain free amino acids (Wang, 2016; Zhang et al., 2020a). These challenges are exacerbated by climate change-induced weather extremes, which intensify drought frequency and severity in tea-growing areas.

Strigolactones (SLs), a class of terpenoid lactone phytohormones, play multifaceted roles in plant growth and stress adaptation (Nomura et al., 2024). Initially, SLs were identified as germination stimulants for Striga seeds when isolated from root exudates of cotton (Cook et al., 1966) and a rhizosphere signaling molecule for promoting symbiotic establishment with mycorrhizal fungi (Akiyama et al., 2005). Recent advances have revealed their involvement in shoot branching (Snowden et al., 2005; Umehara et al., 2008), mesocotyl elongation (Hu et al., 2010; Zheng et al., 2020), root development (Kapulnik et al., 2011; Marzec and Melzer, 2018), leaf senescence (Snowden et al., 2005; Yan et al., 2007; Yamada and Umehara, 2015) and stress response, including nutrient deficiency (Santoro et al., 2021; Marro et al., 2022; Gu et al., 2023), drought stress (Min et al., 2019; Xu et al., 2023; Li et al., 2024), extreme temperature (Omoarelojie et al., 2020, 2021; Zhang et al., 2020b), salt stress (Kong et al., 2017) and heavy metal stress (Tai et al., 2017; Qiu et al., 2021; Chen et al., 2022). For instance, application of GR24 could maintain photosynthetic efficiency and reduce biomass loss under drought stress in rapeseed (Wan et al., 2020), and upregulating antioxidant enzyme activities to mitigate oxidative damage (Li, 2018; Cheng, 2019; Wang et al., 2021; Xu et al., 2023).

The SL biosynthetic pathway initiates with all-trans-β-carotene conversion to 9-cis-β-carotene by DWARF27 (D27), followed by sequential cleavage via CAROTENOID CLEAVAGE DIOXYGENASE7 (CCD7/D17) and CCD8/D10 to produce carlactone (CL), a conserved precursor shared by all SLs. The CL is further diversified into various SLs when transported from the plastid to the cytoplasm, possibly depending on plant species. These SL biosynthesis genes exhibit complex regulatory mechanisms in response to drought stress. Aboveground, drought stress significantly induced the expression of *D27* in rice (Haider et al., 2018) and *FaD27* in tall fescue (Zhuang et al., 2017), while upregulated the expression of *SlCCD7* and *SlCCD8* in tomato (Visentin et al., 2016). These differential responses suggest complex regulatory networks influenced by drought intensity, treatment methods, and species-specific adaptations.

In this study, we investigate SL-mediated drought tolerance mechanisms in tea plants through: i) Physiological characterization of GR24-treated plants under drought stress in hydroponics; ii) Comparative analysis of four cultivars with varying drought tolerance in pot experiments; iii) Overexpression of *CsD27*in Arabidopsis to validate SL function. Our findings demonstrated that SL application alleviated drought-induced physiological damage and revealed a positive correlation between *CsD27* expression levels and drought tolerance across cultivars. These results provided valuable insights into SL-mediated drought resistance mechanisms while establishing a foundation for improving tea quality and yield stability under water-limited conditions.

## 2 Methods and materials

### 2.1 Plant materials and treatments

Annual tea plant cuttings (*Camellia sinensis* L. cv. ‘Longjing43’) were pre-cultured hydroponically in a growth chamber (16 h light/8 h dark cycle, 25°C/23°C, and 50% humidity). The nutrient solution containing 15% PEG-6000 was used to simulate drought stress. Plants exposed to PEG were treated with 0, 5, 10 and 30 μM GR24 via foliar spray every 48 hours, ensuring complete coverage but not dripping. One bud and one leaf of tea plant were harvested after 1 day (24 h)with the first treatment for subsequent experiment. After 8 days of treatment, the shoots of tea plants were collected for physiological indicator determination.

Two-year-old seedlings of *Camellia sinensis* L.cv. Shancha NO.1 (SC), Huangjinya (HJY), Longjin43 (LJ43) and Zijuan (ZJ) were grown in the greenhouse of Tea Research Institute of the Chinese Academy of Agricultural Sciences. Tea seedlings with consistent growth were selected and randomly divided into three groups: (1) Control group (CK), no stress and no GR24 treatment; (2) Drought stress group (DS), no watering was applied during the treatment period.; (3) GR24 treatment group (SL), drought treatment with 10 μM GR24 spray application. The CK and DS groups were sprayed with a 3% acetone solution, while the SL group was sprayed with a 10 μM GR24 solution. All treatments were uniformly watered and sprayed with the corresponding solutions, designated as Day 0. Subsequently, the solutions were sprayed every 2 days (48 h) for a total of 10 days. Following 10 days of water deprivation, the shoots were harvested, immediately flash-frozen in liquid nitrogen, and stored at −80°C for subsequent analyses.

Wild-type *Arabidopsis thaliana* (Columbia-0 ecotype, Col-0) and three independent *CsD27*-overexpressing T3 generation homozygous lines (*OE5-9, OE9-2, OE12-1*) were selected and used for further experiments. For the germination assays, the seeds were sown on 1/2MS medium with and without 200 mM mannitol (control). For the drought stress treatment, the 3-weeks-old seedlings of transgenic lines and WT were grown on soil for one week with or without watering (CK treatment and DS treatment, respectively).

### 2.2 Chlorophyll α fluorescence

The OJIP curves of tea plant were measured at 1, 2, 4, 6 and 8 d after exposure to drought stress and foliar application of GR24 with various concentrations. Chlorophyll α fluorescence was measured after a 30-minute dark treatment using FluorPen FP110 portable fluorometer (Photon Systems Instruments, Czech Republic). Formulae and explanation of the fluorescence parameter used in this study are provided in **Supplementary Table 1**.

### 2.3 Determination of malondialdehyde (MDA) and lipid peroxidation

Fresh leaves were rinsed with deionized water to remove surface contaminants and gently blotted dry with tissues. For each treatment group, six leaves were punched by hole puncher (0.5 cm diameter). The leaf discs were transferred into 50 mL conical tubes containing 15 mL of deionized water. The tubes were shaken (200 rpm) for 1 h to measure the conductivity of the solution, recorded as EC1. Subsequently, samples were boiled for 30 min at 100 °C. Followed by cooling to room temperature, the solution was measured and the results were recorded as EC2. The fresh samples were pulverized in liquid nitrogen, and 0.1 g of each samples was accurately weighed to determined the production rate of MDA and H_2_O_2_ by using the detection kits (Suzhou Comin Biotechnology Co., Ltd, Suzhou, China).

### 2.4 Total RNA isolation and qRT-PCR assay

Total RNA was extracted using the RNA prep Pure Plant kit (TIANGEN biotech CO., Ltd, Beijing, China) and 2 μg of total RNA was used for first-strand cDNA synthesis by the PrimeScript™ RT Reagent Kit (TaKaRa, Dalian). Subsequently, quantitative PCR was performed using SYBR^®^ Premix Ex Taq™ (TaKaRa, Dalian). Transcript levels were normalized to GAPDH, and the calculation of relative transcript abundance using the 2^−ΔΔCt^ method. For each treatment, three biological replicates were used to analyze the expression of each gene.

### 2.5 Subcellular localization

The CDS of *CsD27* (excluding the termination codon) were PCR-amplified and cloned into the pBWA(V)HS-GFP fusion vector. The recombinant constructs were introduced into *A. tumefaciens* through electroporation, followed by transient transformation of *N. benthamiana* leaf epidermis using syringe infiltration. An empty vector control (35S:GFP) was co-infiltrated under identical conditions. At 2 days post-infiltration, transformed leaf sections were excised and mounted on glass slides to detect the subcellular localization of the CsD27 protein using a confocal laser-scanning microscope (Nikon C2-ER).

### 2.6 Determination of catechins

The samples were lyophilized with FreeZone freeze dryer (LABCONCO, America) for 4 days and ground with a RETSCH MM400 grinder (Duesseldorf, Germany). To analyze catechin concentrations, 0.1000g of each freeze-dried samples was used to extract following a high-performance liquid chromatography (HPLC) –based method as previously described in detail (Tong et al., 2023).

## 3 Result

### 3.1 Effects of exogenous GR24 on physiological indexes of tea plant under drought stress

#### 3.1.1 Photosynthetic performance

**Figure 1:**
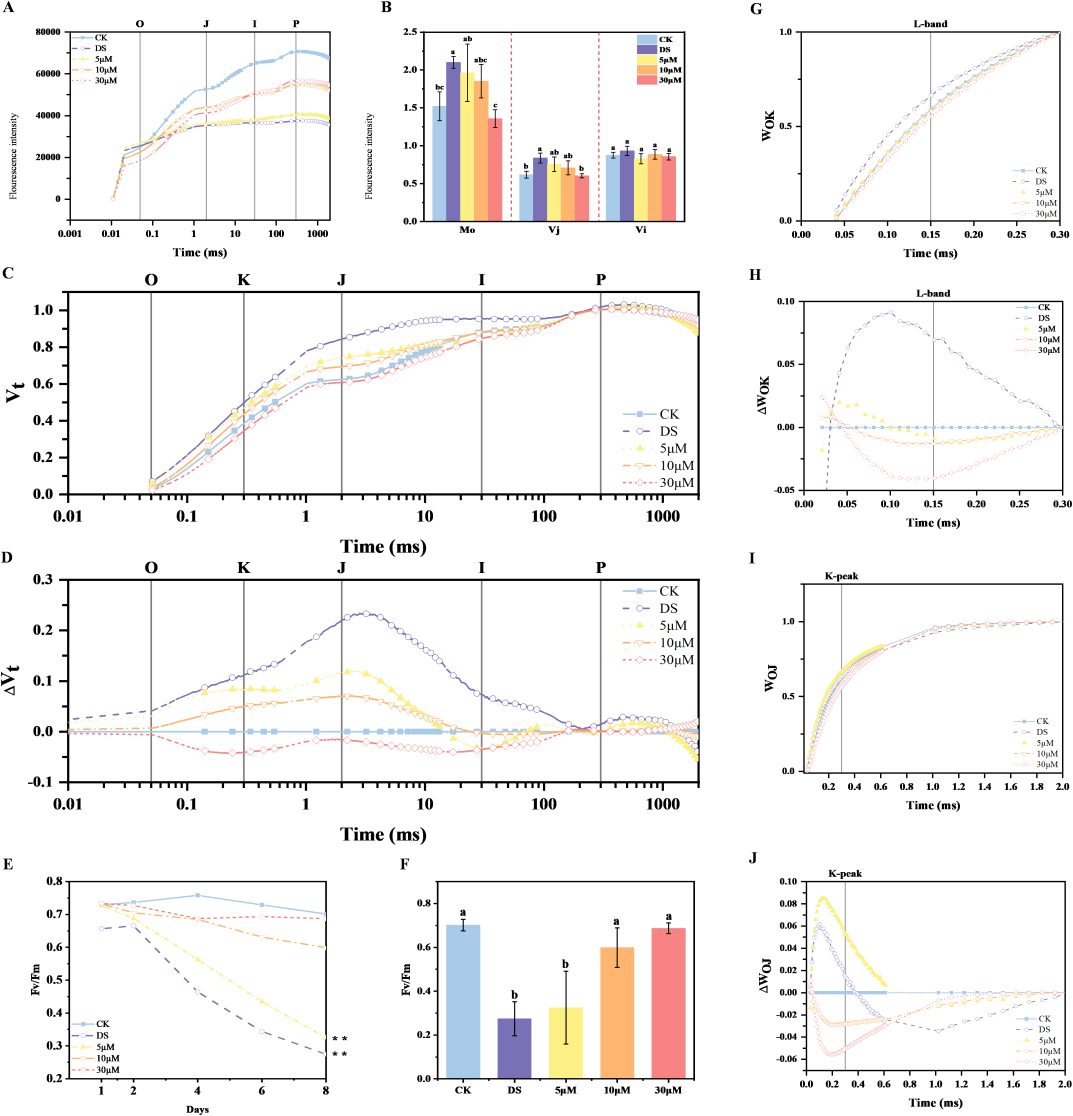
Effects of drought and GR24 treatment on relative fluorescence. **(A)** OJIP curve (on a logarithmic time scale) on day 8, representing the photochemical efficiency of PSII; **(B)** The value of M_0_, V_j_ and V_i_ in each treatment on day 8; **(C)** OJIP curve on day 8 normalized by F_0_ and F_m_ as V_t_ = (F_t_ – F_0_)/(F_m_ – F_0_); **(D)** ΔV_t_ = V_t(treatment)_ – V_t(control)_; **(E)** Trend analysis of Fv/Fm after 8 days of treatment with 15% PEG-6000; **(F)** The value of Fv/Fm on day 8; **(G)** OJIP curve on day 8 normalized by F_0_ and F_k_ as W_OK_ = (F_t_ – F_0_)/(F_K_ – F_0_); **(H)** ΔW_OK_ = W_OK (treatment)_ − W_OK (control;_ **(I)** OJIP curve on day 8 normalized by F_0_ and F_J_ as W_OJ_ = (F_t_ – F_0_)/(F_J_ – F_0_); **(J)** ΔW_OJ_ = W_OJ (treatment)_ − W_OJ (control)._ In **(B, F)**, the values are means ± SD (n > 3), and one-way ANOVA was performed for the statistical analysis. Different letters represent significant differences between treatments (*P* < 0.05). CK, control with no stress; DS, drought stress treatment, 5 μM -30 μM, drought stress treatment with various concentrations of GR24 applied.

OJIP original kinetics were analyzed in CK, DS, and GR24-treated tea plants (5 μM, 10 μM, 30 μM) on day 8, to characterize drought-induced photosynthetic damage, revealing treatment-dependent divergence in fluorescence transients (**Figure 1A**). Compared to CK, drought stress significantly reduced the fluorescence curve and caused the J-step to approach F_p_, leading to the disappearance of the IP phase of the OJIP curve. However, the application of GR24 alleviated the negative effect of drought stress, as indicated by the increase in the fluorescence yield of OJIP curve.

For detailed analysis, fluorescence curves were double-normalized between F_o_ and F_m_ to derive relative variable fluorescence (V_t_) and ΔV_t_ at a logarithmic time scale (**Figures 1C, D**). Upon drought stress, tea plants showed a positive ΔV_t_ amplitude with a distinct J peak, indicating significant impairment of the early stages of electron transfer. As the concentration increased from 0 to 30 μM, the amplitude was gradually decreased and eventually became negative, indicating alleviation of drought-induced impairment in photosynthetic performance.

To obtain further insights into the OJIP curve, additional normalizations and corresponding curve subtractions were performed to gain the L-band and K peak features, reflecting the OK and OJ phases respectively (**Figures 1G-J**). Drought treatment increased both the L-band and the K peak, while GR24 treatments, especially at 10 and 30 μM, reduced these drought-induced increases, demonstrating the positive role of GR24 in improving energetic connectivity and preserving activity of oxygen-releasing complex (OEC) centers under drought stress. The alterations in M_o_ and V_j_ further corroborated these findings (**Figure 1B**). Notably, the K peak of plants treated with 5 μM GR24 under drought stress exceeded that observed in DS treatment, suggesting potential concentration-dependent effects.

Under stress-free conditions, F_v_/F_m_ values in plants typically range from 0.7 to 0.8 (Maxwell and Johnson, 2000), but decline when subjected to environmental stress. Here, we monitored F_v_/F_m_, as a critical indicator of PSII maximum quantum efficiency, after 1, 2, 4, 6 and 8 days(d) of drought stress treatment. The F_v_/F_m_ values of the second leaf of shoot decreased with the prolonged duration of drought stress, however, the application of exogenous GR24 significantly mitigated the damage of photosynthetic apparatus from drought stress (**Figures 1E, F**). Among them, the F_v_/F_m_ values of GR24 at 10 μM and 30 μM concentrations increased by 118.1% and 158.1%. respectively, compared to DS treatment (**Figure 1F**). Taken together, exogenous GR24 effectively mitigates drought-induced damage on photosynthesis of tea plants.

#### 3.1.2 Cell membrane damage

**Figure 2:**
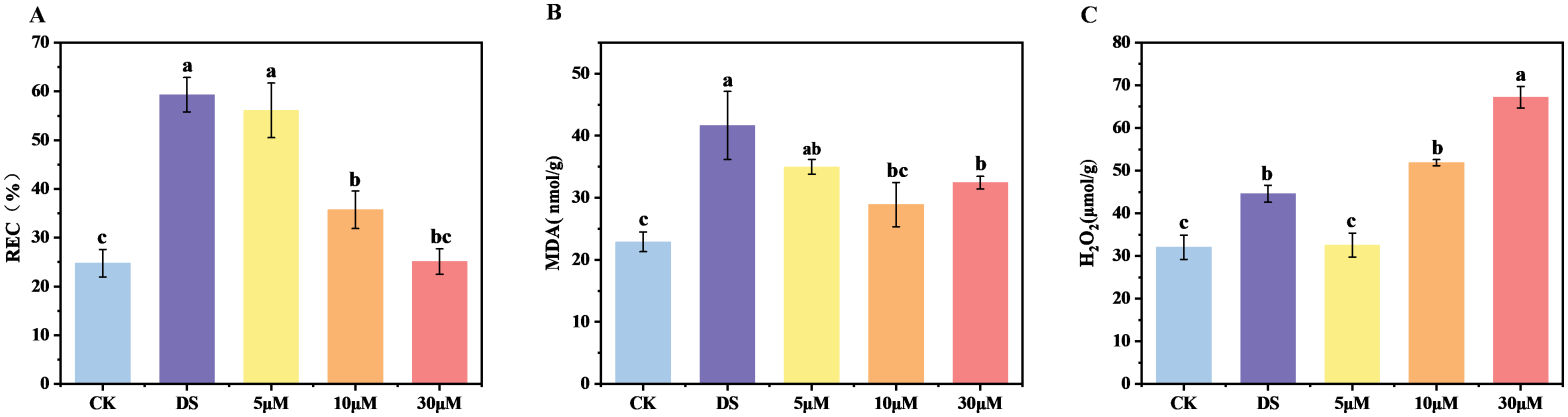
GR24 promoted the drought tolerance of tea plants grown in hydroponics. **(A)** Determination of relative electrical conductivity (REC); **(B)** Determination of malondialdehyde (MDA); **(C)** Determination of H_2_O_2_. In **(A–C)**, values are means ± SD (n > 3), and one-way ANOVA was used to assess statistical differences, where different letters indicate significant differences between treatments (*P* < 0.05). CK, control with no stress; DS, drought stress treatment, 5 μM -30 μM, drought stress treatment with various concentrations of GR24 applied.

Water deficit typically damages the integrity of the cell membrane and lipid peroxidation (Su et al., 2025), as demonstrated by a substantial increase in the levels of relative electrical conductivity (REC) and malondialdehyde (MDA) content. We measured the physiological indicators of each treatment after 8 days of treatment. Compared to CK, the REC and MDA content under drought stress increased significantly by 139.43% and 81.93%, respectively (**Figures 2A, B**). Conversely, all the treatments with different concentrations of GR24 under drought stress, particularly those with 10 μM and 30 μM, mitigated these increases, showing significant reductions of 39.76% and 57.70% in REC, and 19.26% and 9.39% in MDA content, respectively, compared to DS treatment. These physiological results are consistent with the trend observed in chlorophyll fluorescence, further providing evidence for the positive regulation of GR24 on drought tolerance of tea plants.

H_2_O_2_ serves as a critical intracellular signaling molecule in plants. Moderate accumulation of reactive oxygen species (ROS) acts as a prerequisite for triggering plant adaptive mechanisms in response to abiotic stress, while excessive ROS accumulation induces oxidative damage, leading to physiological and metabolic disorders that impair plant growth. Drought stress significantly increased H_2_O_2_ accumulation, reaching approximately 1.3-fold of CK group (**Figure 2C**). However, the effect of exogenous GR24 on H_2_O_2_ accumulation differed from that observed for REC and MDA contents. It was observed that the H_2_O_2_ level was significantly reduced by 26.99% in tea plants treated with 5 μM GR24, but increased by 16.35% and 50.72% in 10 μM and 30 μM treatments, respectively.

### 3.2 The effects of GR24 on different tea plant cultivars under drought stress

**Figure 3:**
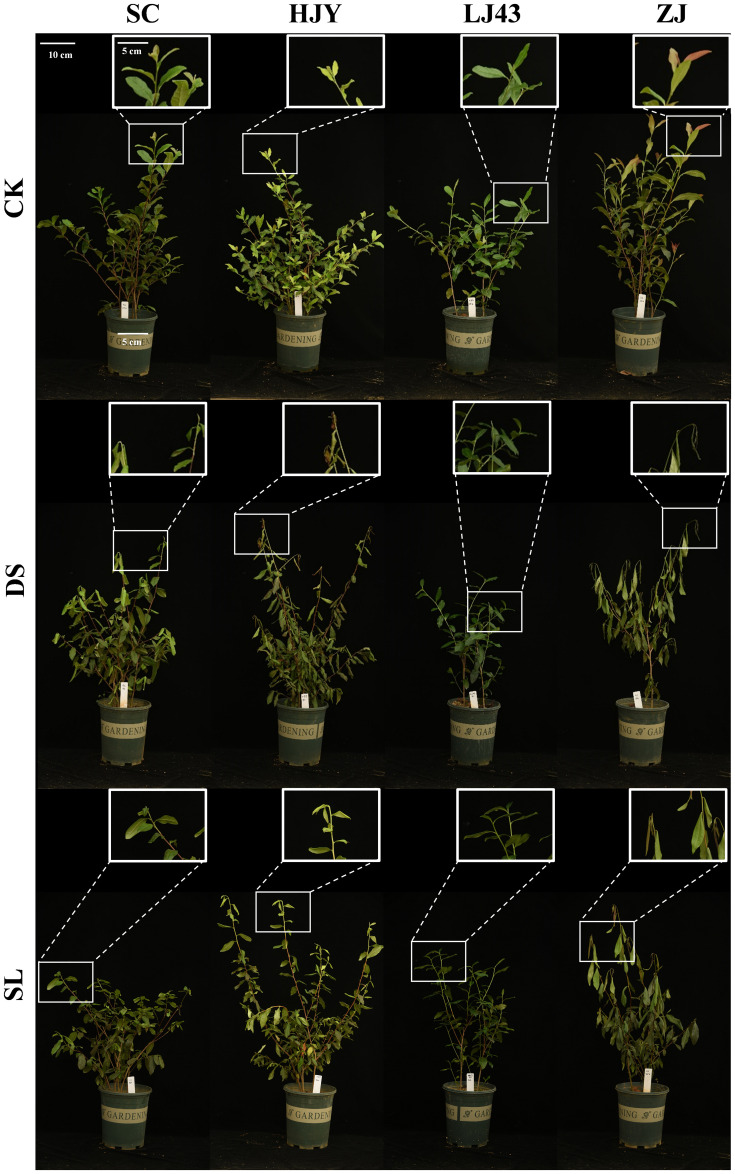
The phenotypic observation of four cultivars after 10 days of drought and GR24 treatment. CK, control with no stress; DS, drought stress treatment; SL, drought stress treatment with 10 µM GR24 applied, scale bar = 10 cm.

To further investigate the relationship between SL and drought tolerance in tea plants, we examined the phenotypic characteristics of four cultivars and measured the MDA content of four cultivars after 10 days drought stress and GR24 treatments (**Figure 3**, **Supplementary Figure 1**), revealing different levels of stress response. SC and LJ43 exhibited milder wilting and lower MDA content, while ZJ showed moderate wilting and MDA levels. In contrast, HJY suffered the most severe damage from drought stress, characterized by burning symptoms appearing on wilted shoots and the highest MDA content.

**Figure 4:**
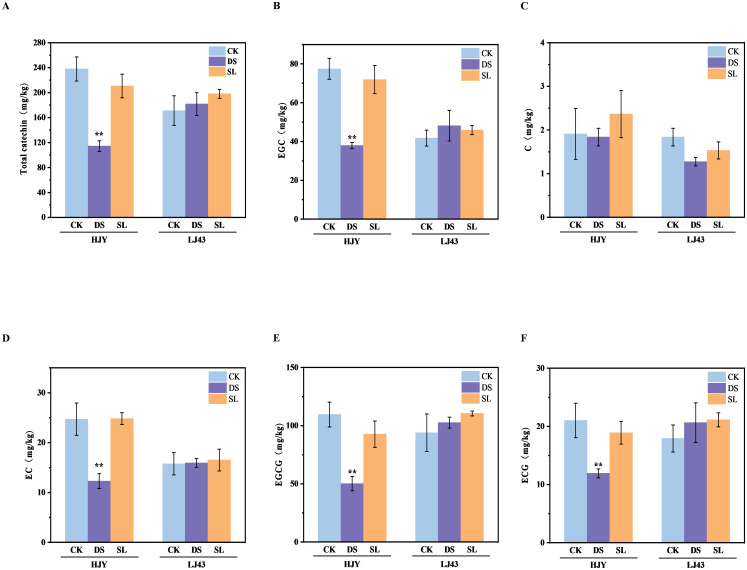
The total and individual catechin content of four cultivars after 10 days of drought and GR24 treatment. **(A)** Total catechin content; **(B)** Epigallocatechin (EGC); **(C)** Catechin (C); **(D)** Epicatechin (EC); **(E)** Epigallocatechin gallate (EGCG); **(F)** Epicatechin gallate (ECG). Values are means ± SD (n > 3). CK, control with no stress; DS, drought stress treatment; SL, drought stress treatment with 10 μM GR24 applied. Two-way ANOVA was used to assess statistical differences, where ** indicates extremely significant differences between treatments and cultivars (*P* < 0.01).

To confirm the impact of SL on tea catechins, the main flavonoid antioxidants, we measured the catechin components in the shoots of drought-insensitive cultivar LJ43 and drought-sensitive cultivar HJY under drought stress (**Figure 4**). Generally, epigallocatechin gallate (EGCG) was the most abundant catechin, followed by epigallocatechin (EGC). In the drought-insensitive cultivar which showed no visible symptoms of drought stress, there were no significant differences in catechin components across the three treatments. However, HJY showed a significantly decrease in both total catechin and individual catechin components. Consistent with the phenotypic observations and MDA content, the application of GR24 alleviated drought-induced damage and promoted catechin accumulation, indicating that SL positively influenced tea quality under drought stress.

### 3.3 CsD27 was significantly correlated to drought tolerance

**Figure 5:**
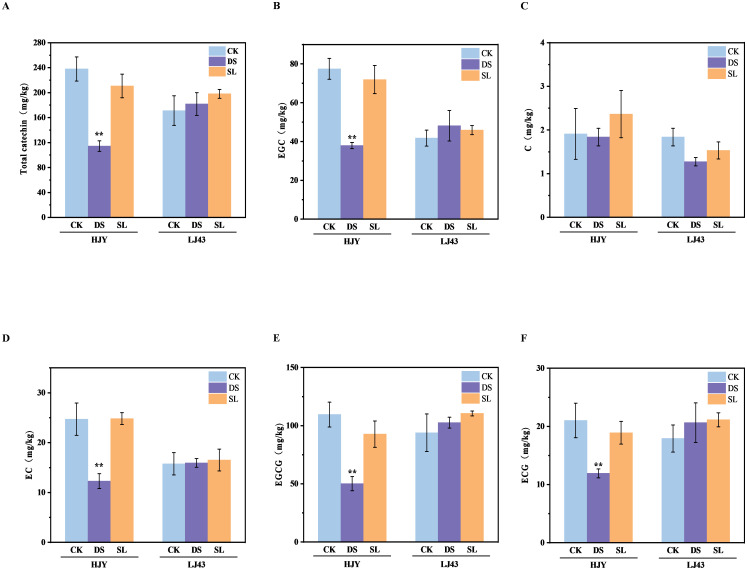
Sequences analysis, subcellular localization and expression patterns of *CsD27*. **(A)** Phylogenetic analysis of *CsD27* with other *D27* genes from other species; **(B)** Expression of *CsD27* in four cultivars without stress; **(C)** Correlation between expression of *CsD27* and other physiological indexes under control **(c)** and drought stress (S) treatments; **(D)** Amino acid sequence analysis of *CsD27* and other *D27* genes from other species; **(E)** Subcellular localization of CsD27 protein through transient expression in tobacco leaf epidermis, scale bar = 20 μm; **(F)** Expression levels of *CsD27* in different tea plant tissues; **(G)** Expression analysis of *CsD27* after 1 day(24 h) treatment. CK, control with no stress; DS, drought stress treatment, 5 μM -30 μM, drought stress treatment with various concentrations of GR24 applied. In **(A, F, G)**, one-way ANOVA was used for statistical analysis, where different letters indicate significant differences between treatments (*P* < 0.05), and and ** indicates extremely significant differences between treatments and cultivars (*P* < 0.01). The values are means ± SD (n > 3).

D27 has been confirmed to be involved in the plant response to abiotic stress across multiple species. Previous studies have shown that the drought resistance of D27 knockout mutants is significantly reduced, while overexpressing lines exhibit the opposite result (Haider et al., 2018). Given the central role of SL in drought response based on the above observation, we identified 8 *CsD27*, the key SL biosynthesis genes, from tea plant genome of *Camellia sinensis* L.cv. Shuchazao (**Supplementary Figure 2**). Specifically, the member (CSS0025398.1) was regulated in *Camellia sinensis* L.cv. Tieguanyin by long-term drought stress as revealed in previous database (unpublished). Additionally, we measured the expression levels of *CsD27* across four tea plant cultivars. While *CsD27* exhibited higher expression levels in SC and LJ43 (drought-tolerant cultivars), its expression was significantly reduced in both HJY and ZJ (drought-sensitive cultivars) (**Figure 5B**). Correlation analysis showed a significant and negative correlation between expression of *CsD27* and MDA content, indicating that cultivars with higher expression levels exhibited higher drought tolerance (**Figure 5C**). Therefore, we cloned the *CsD27* gene (CSS0025398.1) and performed a multiple sequence alignment, followed by the construction of a phylogenetic tree (**Figures 5A, D**). The results demonstrated that *CsD27* shared conserved regions with D27 proteins from other species, and exhibited the highest similarity to *SlD27* and *GmD27*.

### 3.4 Subcellular localization of CsD27

In order to identify the subcellular localization of CsD27 protein, the 35S:*CsD27-GFP* plasmid was introduced into tobacco leaves. The results showed that strong GFP fluorescence of the 35S:CsD27-GFP fusion protein was predominantly observed in the chloroplast, indicating that CsD27 is mainly localized in the chloroplast, consistent with the predicted localization (**Figure 5E**).

### 3.5 Expression patterns of CsD27 in tea plant

To investigate the tissue-specific expression pattern of *CsD27*, total RNA from various tea plant tissues including root, stem, mature leaves, the first leaf of shoot and bud were extracted and used for qPCR (**Figure 5F**). The results showed that the expression level of *CsD27* was higher in mature leaves than other tissues, while exhibited extremely low expression levels in both buds and stems.

Meanwhile, *CsD27* was differentially expressed under stress and hormone treatment. The expression of *CsD27* after 24 hours of DS treatment slightly decreased compared to CK in LJ43. Under GR24 treatment, the expression of *CsD27* transcript was effectively upregulated, with a 2.2-fold increase at 5 μM, a maximum induction of 2.4-fold at 10 μM, and an increase of approximately 1.7-fold at 30 μM (**Figure 5G**). These results implied that *CsD27* might be a crucial gene in response to drought, and the regulation networks were probably related to GR24 concentration.

### 3.6 Overexpression of CsD27 increased the drought tolerance of *Arabidopsis thaliana*

**Figure 6:**
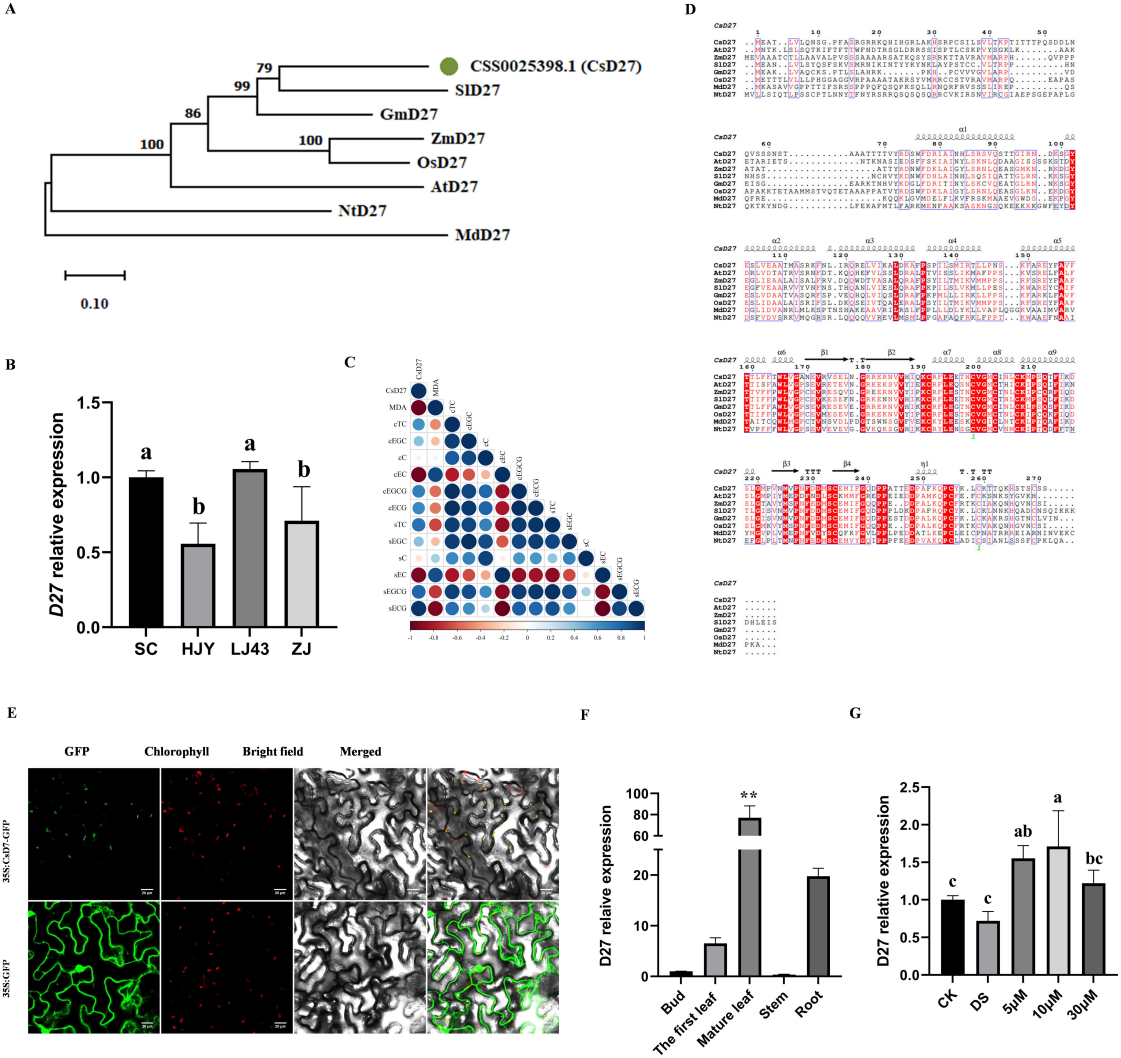
Overexpression of *CsD27* enhances drought tolerance in transgenic *Arabidopsis thaliana*. **(A)** Relative expression levels of *CsD27* gene in wild-type(Col) and *CsD27-OE* transgenic plants (*OE5-9, OE9-2, OE12-1*); **(B)** Determination of MDA; **(C, D)** The germination and phenotypic observation of wild-type and transgenic plants under osmotic stress(200mM Mannitol), scale bar = 1 cm; **(E, F)** The fresh weight measurement and phenotypic observation of wild-type and transgenic plants after 7 days without watering, scale bar = 5 cm. In **(A, E, G)**, one-way ANOVA was performed for the statistical analysis, where different letters represent significant differences (*P* < 0.05), and ** indicates extremely significant differences between treatments and cultivars (*P* < 0.01). In **(B)**, two-way ANOVA was used for the statistical analysis, where different letters represent significant differences (*P* < 0.05). In **(A–C, E)**, values are means ± SD (n > 3). CK, control with no stress; DS, drought stress treatment.

To further investigate its function in drought stress tolerance, we developed three transgenic *Arabidopsis* lines with *CsD27* overexpression (*OE5-9*, *OE9-2*, *OE12-1*). The result indicated that the expression levels of *CsD27* were significantly higher in the transgenic lines than in the wild-type (WT) (**Figure 6A**, **Supplementary Figure 3**).

Seeds of each genotype were planted on 1/2 Murashige and Skoog (MS) media containing 0 or 200 mM mannitol to assess the seed germination rates under osmotic stress. Although germination and cotyledon greening rates were comparable between WT and transgenic lines under 200 mM mannitol treatment, the transgenic lines exhibited larger cotyledon areas and enhanced growth performance compared to WT seedlings under osmotic stress conditions (**Figures 6C, D**). Additionally, we selected three-week-old seedlings of each genotype to expose to one week of drought stress to investigate the role of *CsD27* in drought response of *Arabidopsis*. Compared to WT, the majority of overexpression line seedlings retained green coloration and exhibited better growth under drought stress, as evidenced by less severe wilting phenotype and significantly higher fresh weight (**Figures 6E, F**). Similarly, the MDA result was demonstrated that *OE5-9*, *OE9–2* and *OE12–1* exhibited significant reduction of drought-induced damage, which was consistent with the phenotypic observation (**Figure 6B**). After 3 days of re-watering, the *OE5-9*, *OE9–2* and *OE12–1* seedlings exhibited superior recovery, whereas WT seedlings showed only 25% survival rates (**Figure 6C**). These results indicated that *CsD27* enhanced the drought tolerance of *Arabidopsis*.

## 4 Discussion

Drought stress has emerged as a critical constraint to agricultural productivity worldwide, which has caused billions of economic losses over the past decade (Gupta et al., 2020). The intensification of drought events due to climate change scenarios necessitates ​the development of drought-resistant crop varieties capable of ​maintaining photosynthetic efficiency under ​water-deficit conditions. ​Our study demonstrated that exogenous application of ​SL analog GR24 effectively ​mitigated drought-induced photosynthetic impairment and modulated antioxidant metabolism in tea plants, ​providing new insights into phytohormone-mediated stress adaptation mechanisms.

### 4.1 Photosynthetic apparatus protection by GR24

Water deficit ​induces multilevel photosynthetic dysfunction, ​manifested through stomatal closure, ​structural disorganization of thylakoid membranes, disruption of photosynthetic pigments, and degradation of photosynthetic proteins (Vanlerberghe et al., 2016; Razi and Muneer, 2021). The ΔV_t_ in tea plant revealed that drought stress induced the emergence of a distinct phase in the OJIP kinetic curve, the J peak (**Figure 1D**) caused by the accumulation of Q_A_^-^ in PSII reaction centers (RCs), indicating that drought stress disrupted the electron flow beyond Q_A_ (Guo et al., 2020; Tsimilli-Michael, 2020; Jiang et al., 2025). A significant increase in M_o_ and V_j_ under drought stress further corroborated this result (**Figure 1B**). Whereas, the GR24-treatments efficiently improved the probability of PSII photosynthetic electron transport, thereby conferring protection against drought in the acceptor-side of PSII. Moreover, this effect became more pronounced with increasing GR24 concentrations. The L-band analysis revealed a positive L-band under drought stress, indicating reduced energetic connectivity and system stability (Antunović Dunić et al., 2023). Conversely, GR24 treatment enhanced PSII unit connectivity (**Figures 1G, H**), ​implying ​optimized excitation energy distribution (Yusuf et al., 2010).

Significantly, ​we identified ​a drought-induced K-band ​appearance at 150 μs (**Figures 1I, J**), ​indicative of OEC ​dysfunction (Strasser et al., 2004). Tea plants with 10 μM and 30 μM GR24 had negative K-band, indeed improving the fraction of the active OEC centers. However, the K-band of 5 μM treatment exceeded that of DS treatment. This phenomenon may be caused by SL-induced stomatal closure via ABA-dependent/independent ways, which exacerbated CO_2_ limitation and thereby amplified oxidative stress on the PSII donor side (Cui, 2017; Korek and Marzec, 2023; Zhang et al., 2024). In addition, the results demonstrated that drought stress caused a decline in F_v_/F_m_, while exogenous application of SL mitigated this reduction, indicating enhanced PSII functionality and alleviation of photoinhibition. This findings align with previous reports in wheat (Wei, 2021), grapevine (Min et al., 2019) and *purpureum* (Li et al., 2022), ​suggesting ​conserved SL functions ​across plant taxa.

### 4.2 Oxidative stress modulation and membrane protection

The plant cell membrane plays a vital role in maintaining normal metabolic activities. Our results demonstrated that drought-induced redox imbalance triggered substantial membrane damage in both hydroponic and pot experiments (**Figures 2**, **3**, **Supplementary Figure 1**), consistent with the findings in grapevine (Wang et al., 2021) and soybean (Xie et al., 2024). GR24 treatment ​attenuated damage to cell integrity and oxidative stress with significant reduction in REC and MDA content, which might be attributed to ROS-scavenging capacity ​through ​both expression and activities of antioxidant enzymes (Wang et al., 2021; Xie et al., 2024). Intriguingly, we observed a concentration-dependent dichotomy in H_2_O_2_ regulation (**Figure 2C**), whereby low-dose GR24 suppressed H_2_O_2_ accumulation, while high-dose treatment potentiated ROS signaling. This dual functionality mirrors the hormetic effect of phytohormone signaling, where low-dose GR24 attenuates acute oxidative damage by limiting ROS accumulation, thereby maintaining membrane integrity. In contrast, elevated H_2_O_2_ levels under high-dose GR24 may reflect an adaptive signaling phase, potentially involving ABA-mediated activation of respiratory burst oxidase homologs (RBOHs) (Rodrigues and Shan, 2022). Moreover, the possibility that SLs directly promote ROS production through ABA-independent pathways cannot be excluded (Lv et al., 2018; Wei, 2021). These dual roles of GR24 highlight its complex involvement in redox regulation and drought adaptation, necessitating complementary analyses of antioxidant system activity and ABA biosynthesis profiles.

### 4.3 CsD27 in drought resistance regulation

*D27* is involved in abiotic stress resistance. For example, overexpression of *D27* could decrease drought tolerance in transgenic rice (Haider et al., 2018). A correlation analysis was made to investigate the relationship between *CsD27* and drought tolerance. We found that the cultivars with higher expression levels exhibited better drought tolerance compared to those with lower expression. In *Arabidopsis*, SL-deficient mutants which have a 70–75% reduction in SL content exhibited a hypersensitivity to drought, and they could be rescued when sprayed with SL to almost the same level of WT plants (Ha et al., 2014). Both of these results implicated the involvement of SL as a positive regulator in abiotic stress responses.

Phylogenetic analysis showed that *CsD27* had the closest relationship with *SlD27* and *GmD27* (**Figure 5A**). It was reported that *GmD27* could reduce the accumulation of ROS in an ABA–dependent manner (Han et al., 2025), providing phylogenetic evidence for *CsD27*’s putative role in drought adaptation. To verify this hypothesis, we generated *CsD27*-overexpressing *Arabidopsis* lines (*OE5-9, OE9–2 and OE12-1*). It was found that the transgenic plants with overexpression of *CsD27* had enhanced osmotic resistance for the better growth, lower MDA content and higher survival rate under stress (**Figure 6**). This might contribute to the crucial role for D27 in determining ABA and SL content (Haider et al., 2018). Future investigations should prioritize expanding genetic diversity across tea cultivars to systematically characterize the functional association between *CsD27* allelic variation and drought resistance phenotypes.

### 4.4 Secondary metabolism and tea quality preservation

Catechins are the main flavonoid antioxidants in tea plants, functioning primarily by donating hydrogen atoms and electrons to scavenge ROS (Lv et al., 2021). In the drought-sensitive cultivar HJY, both the total catechins and individual catechin levels declined under drought stress, whereas ​the drought-insensitive cultivar LJ43 ​maintained catechin content, particularly the ​key antioxidants epigallocatechin gallate (EGCG) and epigallocatechin (EGC). Such cultivar-specific response might ​align with ​PAL-mediated ​phenylpropanoid flux redirection under mild drought stress (Chakraborty et al., 2002). Additionally, GR24 application enhanced both total and individual catechin accumulation under drought conditions, suggesting that SL contribute to improved non-enzymatic antioxidant capacity, consistent with the results in crab apple (Xu et al., 2023). Notably, we observed that the expression of *CsD27* was significantly negatively correlated with epicatechin (EC). This correlation may reflect complex SL-mediated effects on isomerization processes and branch-point regulation within the catechin biosynthesis pathway. Future studies are warranted to elucidate the precise role of SL in catechin metabolism and to explore strategies for optimizing catechin synthesis and accumulation through modulation of SL signaling. Moreover, the results also indicated that SLs application under drought stress contributed to tea quality. By preserving catechin levels, SLs not only directly enhanced the catechin accumulation, but also facilitated the production of downstream quality-related metabolites. Since oxidative catechin are inherently unstable, they might be further oxidized to facilitate ​theaflavins (TFs) and thearubigins (TRs), which are critical for taste, liquor color, and aroma quality of black tea (Lv et al., 2021). However, more evidence and experiments are needed to delineate SL-regulated ​biosynthetic nodes ​in ​catechin metabolism, ​potentially ​enabling ​targeted metabolic engineering.

## 5 Conclusion

In summary, this study elucidates that the application of exogenous SL confers drought resilience of tea plants through photosynthetic maintenance, ROS scavenging via catechin accumulation, and mitigating oxidative membrane damage. Meanwhile, the transcript of *CsD27* was correlated with drought tolerance and its overexpression *Arabidopsis* lines enhanced drought adaptation, achieving more than 88% survival rates versus 25% in wild-type controls, alongside preserved membrane integrity (MDA reduced by 59.99%, 61.70% and 54.71% respectively) and biomass (2.82, 1.91 and 2.08-fold higher fresh weight) under drought conditions. Our findings redefined strigolactones as metabolic coordinators that synchronize drought resilience with quality retention in tea plants, establishing an actionable framework for next-generation plant growth regulators. Furthermore, we functionally validated *CsD27* as a molecular determinant of SL-mediated drought adaptation and identified its variants as precision breeding targets, enabling molecular marker-assisted development of elite drought-insensitive cultivars without compromising catechin biosynthesis.

## Data Availability

The original contributions presented in the study are included in the article/**Supplementary Material**. Further inquiries can be directed to the corresponding authors.

## References

[B1] Ahmed S. Griffin T. S. Kraner D. Schaffner M. K. Sharma D. Hazel M.. (2019). Environmental factors variably impact tea secondary metabolites in the context of climate change. Front. Plant Science. 10. doi: 10.3389/fpls.2019.00939 PMC6702324 31475018

[B2] Akiyama K. Matsuzaki K. Hayashi H. (2005). Plant sesquiterpenes induce hyphal branching in arbuscular mycorrhizal fungi. Nature 435, 824–827. doi: 10.1038/nature03608 15944706

[B3] Antunović Dunić J. Mlinarić S. Pavlović I. Lepeduš H. Salopek-Sondi B. (2023). Comparative analysis of primary photosynthetic reactions assessed by OJIP kinetics in three brassica crops after drought and recovery. Appl. Sci. 13, 3078. doi: 10.3390/app13053078

[B4] Chakraborty U. Dutta S. Chakraborty B. (2002). Response of tea plants to water stress. Biol. Plantarum. 45, 557–562. doi: 10.1023/A:1022377126056

[B5] Chen X. Shi X. Ai Q. Han J. Wang H. Fu Q. (2022). Transcriptomic and metabolomic analyses reveal that exogenous strigolactones alleviate the response of melon root to cadmium stress. Hortic. Plant J. 8, 637–649. doi: 10.1016/j.hpj.2022.07.001

[B6] Cheng H. (2019). Effects of strigolactones on the physiological, biochemical and transcriptome characteristics under drought stress in *Setraria italica* (Taigu: Shanxi Agricultural University).

[B7] Cook C. E. Whichard L. P. Turner B. Wall M. E. Egley G. H. (1966). Germination of witchweed (*Striga lutea Lour.*): Isolation and properties of a potent stimulant. Science 154, 1189–1190. doi: 10.1126/science.154.3753.1189 17780042

[B8] Cui Q. (2017). Analysis of the chloroplast proteome of cucumber leaves under elevated CO2 concentration and drought stress (Taian: Shandong Agricultural University).

[B9] Gu P. Tao W. Tao J. Sun H. Hu R. Wang D.. (2023). The D14-SDEL1-SPX4 cascade integrates the strigolactone and phosphate signalling networks in rice. New Phytol., 239, 673–686. doi: 10.1111/nph.18963 37194447

[B10] Guo Y. Lu Y. Goltsev V. Strasser R. J. Kalaji H. M. Wang H.. (2020). Comparative effect of tenuazonic acid, diuron, bentazone, dibromothymoquinone and methyl viologen on the kinetics of Chl a fluorescence rise OJIP and the MR820 signal. Plant Physiol. Biochem. 156, 39–48. doi: 10.1016/j.plaphy.2020.08.044 32906020

[B11] Gupta A. Rico-Medina A. Caño-Delgado A. I. (2020). The physiology of plant responses to drought. Science 368, 266–269. doi: 10.1126/science.aaz7614 32299946

[B12] Ha C. V. Leyva-González M. A. Osakabe Y. Tran U. T. Nishiyama R. Watanabe Y.. (2014). Positive regulatory role of strigolactone in plant responses to drought and salt stress. Proc. Natl. Acad. Sci. U.S.A. 111, 851–856. doi: 10.1073/pnas.1322135111 24379380 PMC3896162

[B13] Haider I. Andreo-Jimenez B. Bruno M. Bimbo A. Floková K. Abuauf H.. (2018). The interaction of strigolactones with abscisic acid during the drought response in rice. J. Exp. Bot. 69, 2403–2414. doi: 10.1093/jxb/ery089 29538660

[B14] Han Y. Sun Y. Wang H. Li H. Jiang M. Liu X.. (2025). Biosynthesis and signaling of strigolactones act synergistically with that of aba and ja to enhance verticillium dahliae resistance in cotton (*Gossypium hirsutum L.*). Plant Cell Environ. 48, 571–586. doi: 10.1111/pce.15148 39286958

[B15] Hu Z. Yan H. Yang J. Yamaguchi S. Maekawa M. Takamure I.. (2010). Strigolactones negatively regulate mesocotyl elongation in rice during germination and growth in darkness. Plant Cell Physiol. 51, 1136–1142. doi: 10.1093/pcp/pcq075 20498118 PMC2900821

[B16] Jiang S. Lu H. Xie Y. Zhou T. Dai Z. Sun R.. (2025). Toxicity of microplastics and nano-plastics to coral-symbiotic alga (*Dinophyceae Symbiodinium*): Evidence from alga physiology, ultrastructure, OJIP kinetics and multi-omics. Water Res. 273, 123002. doi: 10.1016/j.watres.2024.123002 39709880

[B17] Kapulnik Y. Delaux P.-M. Resnick N. Mayzlish-Gati E. Wininger S. Bhattacharya C.. (2011). Strigolactones affect lateral root formation and root-hair elongation in *Arabidopsis* . Planta 233, 209–216. doi: 10.1007/s00425-010-1310-y 21080198

[B18] Kong C. Ren C. Li R. Xie Z. Wang J. (2017). Hydrogen Peroxide and Strigolactones signaling are involved in alleviation of salt stress induced by arbuscular mycorrhizal fungus in sesbania cannabina seedlings. J. Plant Growth Regul. 36, 734–742. doi: 10.1007/s00344-017-9675-9

[B19] Korek M. Marzec M. (2023). Strigolactones and abscisic acid interactions affect plant development and response to abiotic stresses. BMC Plant Biol. 23, 314. doi: 10.1186/s12870-023-04332-6 37308831 PMC10262459

[B20] Li R. (2018). Effects of exogenous strigolactones on growth and physiology of grapevines under drought stress (Shaanxi: Northwest A&F University).

[B21] Li Y. Li S. Feng Q. Zhang J. Han X. Zhang L.. (2022). Effects of exogenous Strigolactone on the physiological and ecological characteristics of *Pennisetum purpureum Schum.* seedlings under drought stress. BMC Plant Biol. 22, 578. doi: 10.1186/s12870-022-03978-y 36510126 PMC9743734

[B22] Li J. Ren J. Zhang Q. Lei X. Feng Z. Tang L.. (2024). Strigolactone enhances tea plant adaptation to drought and *Phyllosticta theicola petch* by regulating caffeine content via *CsbHLH80* . Plant Physiol. Biochem. 216, 109161. doi: 10.1016/j.plaphy.2024.109161 39378645

[B23] Liu S. (2015). Physiological and molecular mechanisms of tea plant responses to dehydration and rehydration (Hangzhou: Tea Research Institute, Chinese Academy of Agricultural Sciences).

[B24] Lv S. Zhang Y. Li C. Liu Z. Yang N. Pan L.. (2018). Strigolactone-triggered stomatal closure requires hydrogen peroxide synthesis and nitric oxide production in an abscisic acid-independent manner. New Phytol. 217, 290–304. doi: 10.1111/nph.14813 28940201

[B25] Lv Z. Zhang C. Shao C. Liu B. Liu E. Yuan D.. (2021). Research progress on the response of tea catechins to drought stress. J. Sci. Food Agric. 101, 5305–5313. doi: 10.1002/jsfa.11330 34031895

[B26] Marro N. Lidoy J. Chico M.Á. Rial C. García J. Varela R. M.. (2022). Strigolactones: New players in the nitrogen–phosphorus signalling interplay. Plant Cell Environ. 45, 512–527. doi: 10.1111/pce.14212 34719040

[B27] Marzec M. Melzer M. (2018). Regulation of root development and architecture by strigolactones under optimal and nutrient deficiency conditions. Int. J. Mol. Sci. 19, 1887. doi: 10.3390/ijms19071887 29954078 PMC6073886

[B28] Maxwell K. Johnson G. N. (2000). Chlorophyll fluorescence—a practical guide. J. Exp. Bot. 51, 659–668. doi: 10.1093/jexbot/51.345.659 10938857

[B29] Min Z. Li R. Chen L. Zhang Y. Li Z. Liu M.. (2019). Alleviation of drought stress in grapevine by foliar-applied strigolactones. Plant Physiol. Biochem. 135, 99–110. doi: 10.1016/j.plaphy.2018.11.037 30529172

[B30] Nomura T. Seto Y. Kyozuka J. (2024). Unveiling the complexity of strigolactones: exploring structural diversity, biosynthesis pathways, and signaling mechanisms. J. Exp. Bot. 75, 1134–1147. doi: 10.1093/jxb/erad412 37877933

[B31] Omoarelojie L. O. Kulkarni M. G. Finnie J. F. Pospíšil T. Strnad M. Van Staden J. (2020). Synthetic strigolactone (rac-GR24) alleviates the adverse effects of heat stress on seed germination and photosystem II function in lupine seedlings. Plant Physiol. Biochem. 155, 965–979. doi: 10.1016/j.plaphy.2020.07.043 32977141

[B32] Omoarelojie L. O. Kulkarni M. G. Finnie J. F. Van Staden J. (2021). Strigolactone analog (rac-GR24) enhances chilling tolerance in mung bean seedlings. South Afr. J. Bot. 140, 173–181. doi: 10.1016/j.sajb.2021.03.044

[B33] Qiu C.-W. Zhang C. Wang N.-H. Mao W. Wu F. (2021). Strigolactone GR24 improves cadmium tolerance by regulating cadmium uptake, nitric oxide signaling and antioxidant metabolism in barley (*Hordeum vulgare* L.). Environ. pollut. 273, 116486. doi: 10.1016/j.envpol.2021.116486 33484996

[B34] Razi K. Muneer S. (2021). Drought stress-induced physiological mechanisms, signaling pathways and molecular response of chloroplasts in common vegetable crops. Crit. Rev. Biotechnol. 41, 669–691. doi: 10.1080/07388551.2021.1874280 33525946

[B35] Rodrigues O. Shan L. (2022). Stomata in a state of emergency: H_2_O_2_ is the target locked. Trends Plant Sci. 27, 274–286. doi: 10.1016/j.tplants.2021.10.002 34756808

[B36] Santoro V. Schiavon M. Visentin I. Constán-Aguilar C. Cardinale F. Celi L. (2021). Strigolactones affect phosphorus acquisition strategies in tomato plants. Plant Cell Environ. 44, 3628–3642. doi: 10.1111/pce.14169 34414578 PMC9290678

[B37] Snowden K. C. Simkin A. J. Janssen B. J. Templeton K. R. Loucas H. M. Simons J. L.. (2005). The *Decreased apical dominance1/Petunia hybrida CAROTENOID CLEAVAGE DIOXYGENASE8* Gene Affects Branch Production and Plays a Role in Leaf Senescence, Root Growth, and Flower Development. Plant Cell 17, 746–759. doi: 10.1105/tpc.104.027714 15705953 PMC1069696

[B38] Strasser R. J. Tsimilli-Michael M. Srivastava A. (2004). “Analysis of the chlorophyll a fluorescence transient,” in Chlorophyll a Fluorescence: A Signature of Photosynthesis. Eds. Papageorgiou G. C. Govindjee (Springer Netherlands, Dordrecht), 321–362. doi: 10.1007/978-1-4020-3218-9_12

[B39] Su X. Yao L. Wang X. Zhang Y. Zhang G. Li X. (2025). Mechanisms for cell survival during abiotic stress: focusing on plasma membrane. Stress Biol. 5, 1. doi: 10.1007/s44154-024-00195-5

[B40] Tai Z. Yin X. Fang Z. Shi G. Lou L. Cai Q. (2017). Exogenous gr24 alleviates cadmium toxicity by reducing cadmium uptake in switchgrass (*Panicum virgatum*) seedlings. Int. J. Environ. Res. Public Health 14, 852. doi: 10.3390/ijerph14080852 28758909 PMC5580556

[B41] Tong M. Chen X. Bai Y. Zhou P. Feng Z. Lai J.. (2023). Quality characteristics of white leaf tea of ‘Baiye 1’ (*Camellia sinensis*) in different producing areas. Agronomy 13, 2526. doi: 10.3390/agronomy13102526

[B42] Tsimilli-Michael M. (2020). Revisiting JIP-test: An educative review on concepts, assumptions, approximations, definitions and terminology. Photosynthetica 58, 275–292. doi: 10.32615/ps.2019.150

[B43] Umehara M. Hanada A. Yoshida S. Akiyama K. Arite T. Takeda-Kamiya N.. (2008). Inhibition of shoot branching by new terpenoid plant hormones. Nature 455, 195–200. doi: 10.1038/nature07272 18690207

[B44] Vanlerberghe G. C. Martyn G. D. Dahal K. (2016). Alternative oxidase: a respiratory electron transport chain pathway essential for maintaining photosynthetic performance during drought stress. Physiologia Plantarum 157, 322–337. doi: 10.1111/ppl.12451 27080742

[B45] Visentin I. Vitali M. Ferrero M. Zhang Y. Ruyter-Spira C. Novák O.. (2016). Low levels of strigolactones in roots as a component of the systemic signal of drought stress in tomato. New Phytol. 212, 954–963. doi: 10.1111/nph.14190 27716937

[B46] Wan L. Li Z. Li S. Liu L. Ma N. Zhang C. (2020). Alleviation effects of exogenous strigolactone on growth of brassica napus L. seedling under drought stress. Chin. J. Oil Crop Sci. 42, 461–471. doi: 10.19802/j.issn.1007-9084.2019188

[B47] Wang W. (2016). Transcriptome analysis of *Camellia sinensis* under heat and drought stress and functional characterization of Histone Hi gene (Nanjing: Nanjing Agricultural University).

[B48] Wang W. Min Z. Wu J. Liu B. Xu X. Fang Y.. (2021). Physiological and transcriptomic analysis of Cabernet Sauvginon (Vitis vinifera L.) reveals the alleviating effect of exogenous strigolactones on the response of grapevine to drought stress. Plant Physiol. Biochem. 167, 400–409. doi: 10.1016/j.plaphy.2021.08.010 34411779

[B49] Wei R. (2021). Mechanism of alleviation effects of exogenous strigolactones on drought stress in wheat (Hefei: Anhui University).

[B50] Xie F. Liu Y. Zhao Q. Liu X. Wang C. Wang Q.. (2024). Exogenous application of melatonin and strigolactone by regulating morphophysiological responses and gene expression to improve drought resistance in fodder soybean seedlings. Agronomy 14, 1803. doi: 10.3390/agronomy14081803

[B51] Xu J. Li L. Liu Y. Yu Y. Li H. Wang X.. (2023). Molecular and physiological mechanisms of strigolactones-mediated drought stress in crab apple (*Malus hupehensis Rehd.*) seedlings. Scientia Hortic. 311, 111800. doi: 10.1016/j.scienta.2022.111800

[B52] Yamada Y. Umehara M. (2015). Possible roles of strigolactones during leaf senescence. Plants 4, 664–677. doi: 10.3390/plants4030664 27135345 PMC4844400

[B53] Yan H. Saika H. Maekawa M. Takamure I. Tsutsumi N. Kyozuka J.. (2007). Rice tillering dwarf mutant dwarf3 has increased leaf longevity during darkness-induced senescence or hydrogen peroxide-induced cell death. Genes Genet. Syst. 82, 361–366. doi: 10.1266/ggs.82.361 17895586

[B54] Yang H. Xia L. Li J. Jia X. Jia X. Qi Y.. (2024). *CsLAC4*, regulated by *CsmiR397a*, confers drought tolerance to the tea plant by enhancing lignin biosynthesis. Stress Biol. 4, 50. doi: 10.1007/s44154-024-00199-1 39641904 PMC11624182

[B55] Yusuf M. A. Kumar D. Rajwanshi R. Strasser R. J. Tsimilli-Michael M. Govindjee. (2010). Overexpression of γ-tocopherol methyl transferase gene in transgenic *Brassica juncea* plants alleviates abiotic stress: Physiological and chlorophyll a fluorescence measurements. Biochim. Biophys. Acta - Bioenergetics 1797, 1428–1438. doi: 10.1016/j.bbabio.2010.02.002 20144585

[B56] Zhang J. Chen X. Song Y. Gong Z. (2024). Integrative regulatory mechanisms of stomatal movements under changing climate. J. Integr. Plant Biol. 66, 368–393. doi: 10.1111/jipb.13611 38319001

[B57] Zhang C. Wang M. Chen J. Gao X. Shao C. Lv Z.. (2020a). Survival strategies based on the hydraulic vulnerability segmentation hypothesis, for the tea plant [*Camellia sinensis(*L.) O. Kuntze] in long-term drought stress condition. Plant Physiol. Biochem. 156, 484–493. doi: 10.1016/j.plaphy.2020.09.034 33038691

[B58] Zhang X. Zhang L. Sun Y. Zheng S. Wang J. Zhang T. (2020b). Hydrogen peroxide is involved in strigolactone induced low temperature stress tolerance in rape seedlings (Brassica rapa L.). Plant Physiol. Biochem. 157, 402–415. doi: 10.1016/j.plaphy.2020.11.006 33197729

[B59] Zheng J. Hong K. Zeng L. Wang L. Kang S. Qu M.. (2020). Karrikin signaling acts parallel to and additively with strigolactone signaling to regulate rice mesocotyl elongation in darkness. Plant Cell 32, 2780–2805. doi: 10.1105/tpc.20.00123 32665307 PMC7474294

[B60] Zhuang L. Wang J. Huang B. (2017). Drought inhibition of tillering in *Festuca arundinacea* associated with axillary bud development and strigolactone signaling. Environ. Exp. Bot. 142, 15–23. doi: 10.1016/j.envexpbot.2017.07.017

