# Supplementary material for: Exogenous strigolactones modulate antioxidant metabolism via CsD27 to enhance drought tolerance in tea plants
Source: Front Plant Sci. 2025 Jun 6;16:1601094. doi: 10.3389/fpls.2025.1601094 (PMC12179142; doi:10.3389/fpls.2025.1601094)
Supplement: Supplementary file 1 [file Table1.docx]

Supplementary Material

## 1.Supplementary Figures


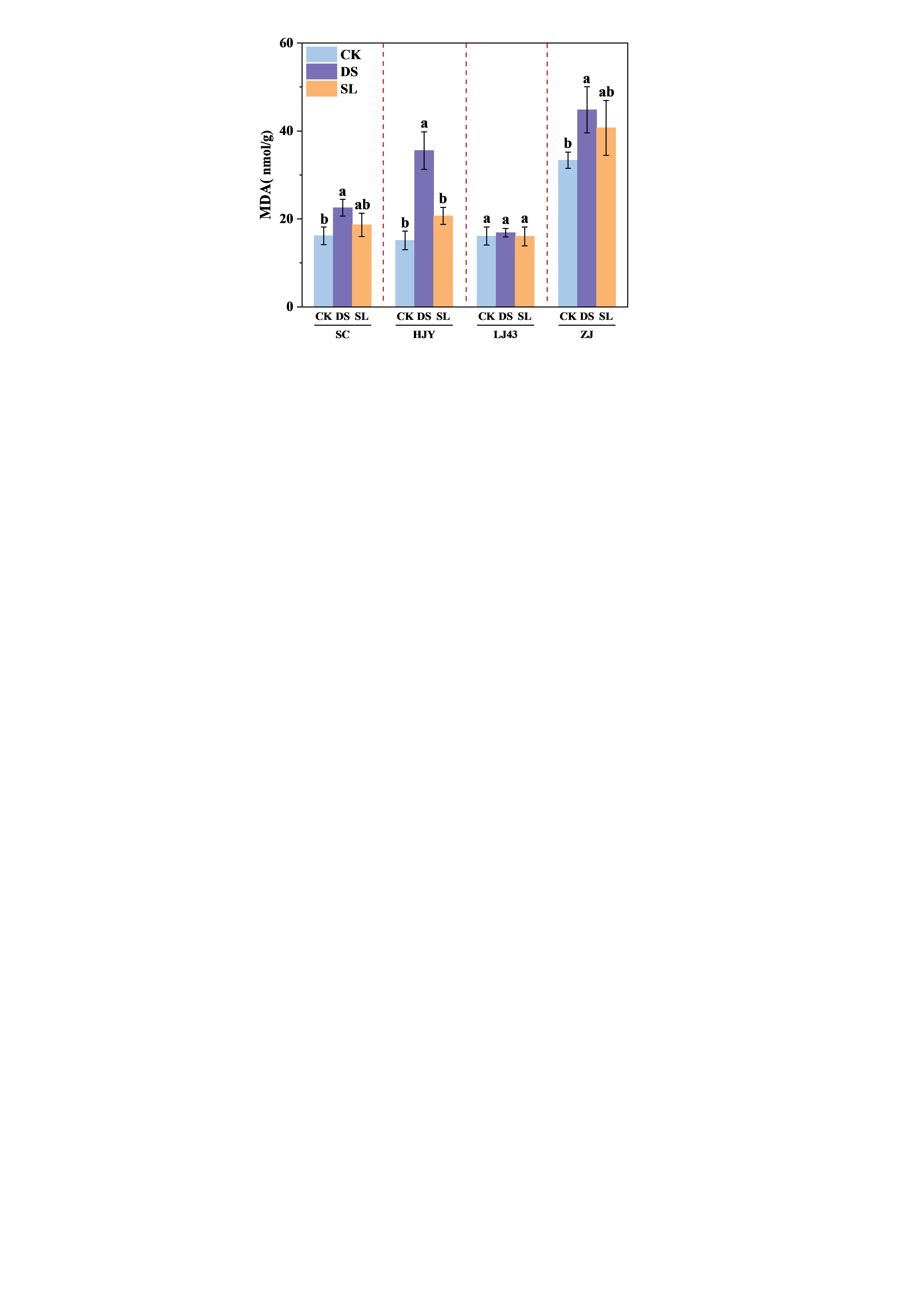


**Supplementary Figure 1.** The MDA content of four cultivars after 10 days of drought and GR24 treatment. CK, control with no stress; DS, drought stress treatment; SL, drought stress treatment with 10 μM GR24 applied. Values are means ± SD (n > 3). One-way ANOVA was used to assess statistical differences, where different letters indicate significant differences between treatments (*P* < 0.05).


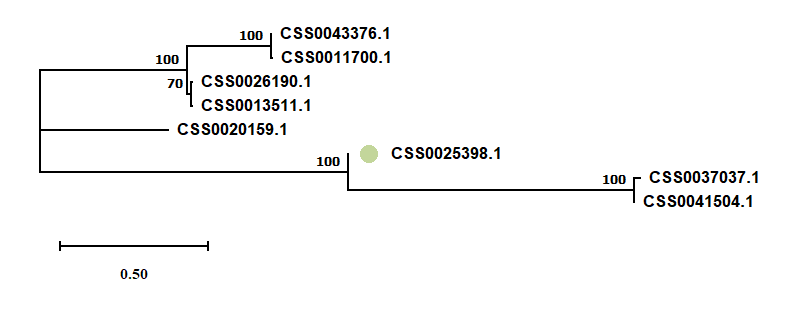


**Supplementary Figure 2.** Phylogenetic analysis of CsD27 family members. The phylogenetic tree was constructed using the neighbor-joining method with bootstrapping with 1,000 iterations by MEGA 7.0.


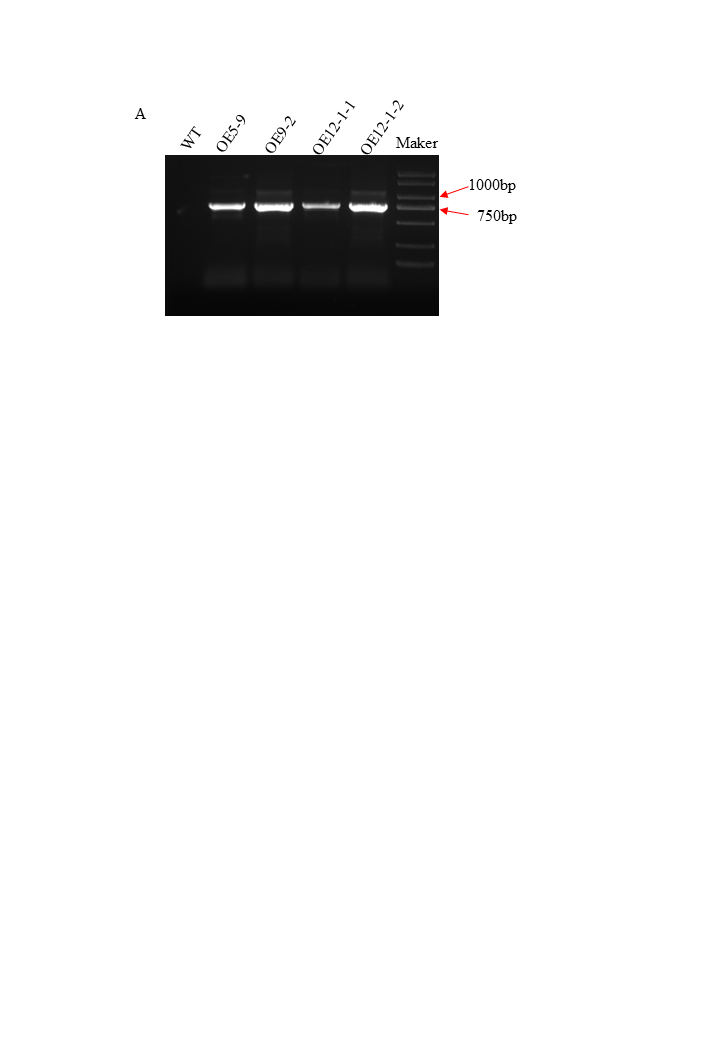


**Supplementary Figure 3.** Identification of CsD27.4-overexpressing plants. Marker: Maker Ⅲ; WT: wild type (negative control); OE5-9,OE-9-2,OE12-1-1,OE12-1-2: *CsD27*-transgenic positive plants.

## 2.Supplementary Table

**Supplementary Table 1.** Formulae and explanation of fluorescence parameter used in this study.

| Fuorescence parameter | Formulae and explanation |
| --- | --- |
| F_0_ | Minimum fluorescence |
| F_50μs_ ≈ F_0_ | Fluorescence intensity at 50 µs |
| F_k_ | Fluorescence intensity at 300 µs |
| F_j_ | Fluorescence intensity at J-step (at 2 ms) |
| F_i_ | Fluorescence intensity at i-step (at 30 ms) |
| F_P_=F_m_ | Maximal fluorescence intensity |
| F_V_ | Maximal variable fluorescence. F_V_=F_m_-F_0_ |
| V_i_ | Relative variable fluorescence at the I-step. V_i_=(F_i_−F_0_)/(F_m_−F_0_) |
| V_j_ | Relative variable fluorescence at the J-step. V_j_=(F_j_-F_0_)/(F_m_-F_0_) |
| M_0_ | M_0_ = TR_0_ /RC−ET_0_/RC = 4(F_300_−F_0_)/(F_m_−F_0_). Initial slope (in ms^−1^) of the fluorescence transient. |
